# Cellars

**Published:** 1873-07

**Authors:** 


### CELLARS.

THERE ought to be no cellar under the dwelling of a civilized man, who wants himself, and family, and servants to live healthfully and long. A cellar usually means a receptacle for old and worthless articles of household use, such as rusted iron and tinware, old boots and shoes, and bones and rotten vegetables and fruits; damp boards, piles of ashes and every conceivable thing which attracts dampness, and mould, and decay. The emanations from all these things fill the air with the most hurtful constituents. This air comes up through the crevices of the floors and other openings and spreads through every room in the house, and engenders obscure diseases, in many cases, and sometimes fatal.

Where there are cellars care should be taken to open the doors or windows for an hour or two every clear day from April to November; during this time the walls and ceilings should be well swept every month, and the whole floor once a week; special pains should be taken to remove everything that can decay after a few days; fresh, unslacked lime should be scattered over dark and damp places once a month. The ceilings of all cellars should be well plastered, and with the walls should be whitewashed every summer.

